# Molecular Basis for Ser/Thr Specificity in PKA Signaling

**DOI:** 10.3390/cells9061548

**Published:** 2020-06-25

**Authors:** Matthias J. Knape, Maximilian Wallbott, Nicole C. G. Burghardt, Daniela Bertinetti, Jan Hornung, Sven H. Schmidt, Robin Lorenz, Friedrich W. Herberg

**Affiliations:** Department of Biochemistry, University of Kassel, 34132 Kassel, Germany; maknape@googlemail.com (M.J.K.); m.wallbott@uni-kassel.de (M.W.); burghardt@uni-kassel.de (N.C.G.B.); d.bertinetti@uni-kassel.de (D.B.); j.hornung@student.uni-kassel.de (J.H.); sven_schmidt@uni-kassel.de (S.H.S.)

**Keywords:** cAMP-dependent protein kinase, PKA, cAMP signaling, protein kinases, kinase function, phosphorylation, substrate specificity, Ser/Thr specificity, surface plasmon resonance

## Abstract

cAMP-dependent protein kinase (PKA) is the major receptor of the second messenger cAMP and a prototype for Ser/Thr-specific protein kinases. Although PKA strongly prefers serine over threonine substrates, little is known about the molecular basis of this substrate specificity. We employ classical enzyme kinetics and a surface plasmon resonance (SPR)-based method to analyze each step of the kinase reaction. In the absence of divalent metal ions and nucleotides, PKA binds serine (PKS) and threonine (PKT) substrates, derived from the heat-stable protein kinase inhibitor (PKI), with similar affinities. However, in the presence of metal ions and adenine nucleotides, the Michaelis complex for PKT is unstable. PKA phosphorylates PKT with a higher turnover due to a faster dissociation of the product complex. Thus, threonine substrates are not necessarily poor substrates of PKA. Mutation of the DFG+1 phenylalanine to β-branched amino acids increases the catalytic efficiency of PKA for a threonine peptide substrate up to 200-fold. The PKA Cα mutant F187V forms a stable Michaelis complex with PKT and shows no preference for serine versus threonine substrates. Disease-associated mutations of the DFG+1 position in other protein kinases underline the importance of substrate specificity for keeping signaling pathways segregated and precisely regulated.

## 1. Introduction

Signaling via the second messenger 3′,5′-cyclic adenosine monophosphate (cAMP) is a common concept of eukaryotic signal transduction. cAMP signaling regulates a myriad of physiological conditions such as the metabolism of glycogen and lipids, long-term potentiation, and endocrine function [1,2]. Furthermore, several human diseases have recently been linked to abnormalities of the cAMP signaling pathway [3]. Binding of a first messenger such as epinephrine to its respective seven-transmembrane G protein-coupled receptor leads to the dissociation of a G_sα_ protein, which in turn activates adenylate cyclases to produce cAMP out of ATP. As a second messenger, cAMP binds to and thus activates effector proteins. The major receptor of cAMP is the cAMP-dependent protein kinase (PKA) [4,5]. In its inactive state, PKA forms a heterotetramer consisting of a regulatory (R) subunit dimer and two catalytic (C) subunits (R_2_C_2_). Binding of two cAMP molecules per R subunit induces conformational changes unleashing the catalytic activity of the C subunits to phosphorylate downstream protein substrates. Besides the R isoforms, the heat-stable protein kinase inhibitors (PKIs, [6]) are high-affinity, physiological pseudosubstrate inhibitors of most C subunits in various organisms. These small proteins can inhibit PKA activity also in the nucleus and export the kinase into the cytoplasm [7].

Protein phosphorylation is an important post-translational modification that affects the activity, stability as well as interactions and cellular localization of proteins. The humane kinome consists of more than 500 protein kinases [8]. Eukaryotic protein kinases are classified into three categories depending on the amino acids they phosphorylate: (1) Ser/Thr kinases, (2) Tyr kinases, and (3) a small group of dual-specific kinases, which can phosphorylate both Ser/Thr and Tyr substrates [9,10]. Additionally, effector proteins that contain phosphosite-specific domains allow for further specificity in cellular signaling. As an example, 14-3-3 proteins are phospho-Ser (pSer)-specific, forkhead-associated (FHA) domains are phospho-Thr (pThr)-specific, while Src homology 2 (SH2) domains specifically bind to phospho-Tyr (pTyr) residues [11,12,13].

**Figure 1:**
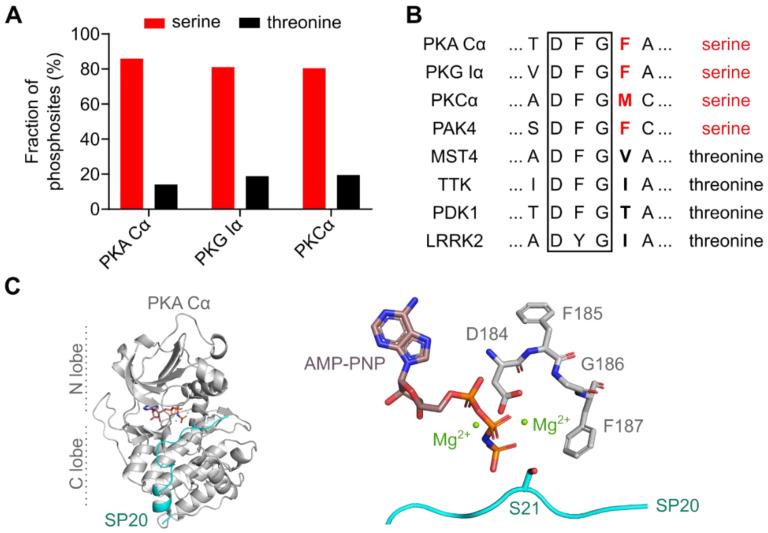
PKA prefers serine over threonine as a phosphoryl acceptor residue. (**A**) The AGC kinases PKA, PKG, and PKC are serine-specific. Bar diagram showing the fraction of annotated phosphosites for serine and threonine phosphoryl acceptors. Data were obtained from the PhosphoSitePlus® database v6.5.9.1 [23]. (**B**) Alignment of the DFG motif (black box) and the DFG+1 residues (bold letters) of human Ser/Thr protein kinases that prefer either serine (red) or threonine (black) as phosphoryl acceptor. The alignment was generated with Clustal Omega [24]. UniProt IDs: P17612 (PKA Cα); Q13976 (PKG Iα); P17252 (PKCα); O96013 (PAK4); Q9P289 (MST4); P33981 (TTK); O15530 (PDK1); Q5S007 (LRRK2). (**C**) Crystal structure of the murine PKA Cα subunit with AMP-PNP, Mg^2+^, and SP20 bound (PDB code: 4DG0) [25]. A zoomed view (right panel) shows the DFG motif (residues 184-186) and the DFG+1 residue (F187) interacting with Mg_2_AMP-PNP and the substrate SP20. S21 is the phosphoryl acceptor residue of SP20. All structure images were generated using the PyMOL Molecular Graphics System (Version 2.2.2; Schrödinger, LLC, New York, NY, USA).

Interestingly, in most cases, Ser/Thr kinases show a preference for either serine or threonine residues (Figure 1A) [14]. The PKA C subunit is a classic example for a Ser/Thr kinase with serine specificity and this preference is highly conserved among different species [15]. PKA can select target substrates according to the consensus sequence RRXS*/T*Y, where X is any residue, and Y is a hydrophobic residue [16,17]. However, the kinase is also capable of phosphorylating substrates that do not exhibit this canonical consensus sequence [15,18], and the consensus motif is less critical for the recognition of protein substrates compared to peptide substrates [19]. The best-established synthetic PKA substrate is Kemptide, a heptapeptide (LRRA**S**LG), and a canonical serine substrate derived from the porcine liver pyruvate kinase [20,21]. Interestingly, PKA phosphorylates a peptide derived from PKI with a serine introduced at the P0 position (GRTGRRN**S**I) with more than 70-fold higher catalytic efficiency than Kemptide [22].

Originally, the lack of identified threonine substrates of PKA in vivo has been attributed to low K_M_ and k_cat_ values for threonine peptides [20]. However, today, some important threonine substrates of PKA have been identified, amongst those the protein phosphatase 1 regulatory subunit 1B (also known as dopamine- and cAMP-regulated neuronal phosphoprotein, DARPP32) [26].

How a Ser/Thr kinase differentiates between the two phosphoryl acceptors that only differ in one methyl group remained unclear for a long time. Chen and coworkers have recently shown that the residue following the highly conserved DFG motif (DFG+1) is the main determinant for Ser/Thr specificity (Figure 1B,C) [27,28]. Kinases that prefer serine as a phosphoryl acceptor carry a large hydrophobic amino acid residue in this position, such as leucine, methionine, or phenylalanine. In contrast, kinases that prefer threonine substrates carry either β-branched or small amino acid residues like isoleucine or valine in the DFG+1 position. Large, i.e., sterically more demanding, DFG+1 residues of serine-specific kinases hinder the binding of threonine substrates, while β-branched amino acids tolerate the additional methyl group of the threonine side chain [28].

Phosphoryl acceptor preference of protein kinases is generally described using basic enzyme kinetics parameters. PKA strongly prefers serine over threonine residues as phosphoryl acceptor, which is indicated not only by 4-fold higher k_cat_ values but also by a more than 30-fold increase in K_M_ for synthetic threonine substrate peptides compared to their serine-containing counterparts [20,29]. Moreover, characterization of the individual steps of the phosphorylation reaction using viscosimetry as well as rapid quench kinetics led to the conclusion that serine- and threonine-containing peptides have comparable affinities to the kinase [29].

Referring to K_M_ values for serine and threonine peptides for PAK4 (p21-activated kinase 4) and MST4 (mammalian STE20-like protein kinase 4) kinases, Chen et al. reasoned that a threonine residue in the P0 position would not affect substrate affinity [28]. However, the Michaelis constant (K_M_) is the substrate concentration at which the half-maximal enzyme activity is achieved, and therefore, K_M_ values refer to the whole catalytic cycle including substrate association and release, phosphoryl transfer, and product release [29]. PKA is a highly dynamic enzyme, and thus, not every catalytic cycle results in substrate phosphorylation leading to varying turnover rates [30]. For the PKA C subunit, the chemical reaction of peptide phosphorylation is fast, while the release of the product ADP is diffusion-controlled, and thus the rate-determining step [31,32]. Accordingly, specific assumptions must be made to equate the K_M_ with the equilibrium dissociation constant (K_D_) which describes (substrate) affinity. In the case of the PKA C subunit, the K_D_ for Kemptide is at least one order of magnitude higher than the K_M_ [33,34]. In this study, we, therefore, aimed to answer the fundamental question, whether the phosphoryl acceptor residue itself influences substrate affinity. We used the Cα subunit of PKA to analyze the phosphorylation and the binding kinetics of PKI-derived protein substrates. In the presence of metal and nucleotide, the serine substrate PKS displays a higher affinity to PKA, when compared to the analogous threonine substrate, PKT. However, PKA phosphorylates PKT faster. The tendency in affinities can be switched by mutation of the DFG+1 F187 to valine in the PKA C subunit (F187V). Thereby we demonstrate that binding affinity towards a substrate is indeed affected by the phosphoryl acceptor residue. Our results suggest that the specificity of a Ser/Thr kinase depends not only on the turnover but also on the substrate affinity.

## 2. Materials and Methods

### 2.1. Protein Preparation

GST fusion proteins were expressed and purified as described earlier [35]. Briefly, coding pGEX-KG plasmids were transformed in *E. coli* BL21 (DE3) cells and expression was induced with 0.4 mM IPTG for 16 h at room temperature. Finally, the fusion proteins were purified using Protino glutathione agarose 4B (MACHEREY-NAGEL, Düren, Germany) according to the manufacturer’s instructions. The threonine substrate GST-PKT (=GST-PKI A21T) was generated by site-directed mutagenesis using the following primer pair: forward: 5′-CGACGTAACACCATCCACGATATCC-3′ and reverse: 5′-GGATATCGTGGATGGTGTTACGTCG-3′.

Constructs of the PKA human Cα isoform (UniProt ID: P17612) were expressed and purified as previously described [36,37]. Recombinant proteins were expressed in T7 Express I^q^ Competent *E. coli* cells (New England Biolabs, Ipswich, MA, United States) for 16 h at room temperature after induction with 0.4 mM IPTG. The DFG+1 mutations F187V, F187I, and F187T were introduced by site-directed mutagenesis using the site-specific primers F187V_forward: 5′-GACTTCGGTGTCGCCAAGCGC-3′ and F187V_reverse: 5′-GCGCTTGGCGACACCGAAGTC-3′, F187I_forward: 5′-GACTTCGGTATCGCCAAGCGC-3′, F187I_reverse: 5′-GCGCTTGGCGATACCGAAGTC-3′, F187T_forward: 5′-GACTTCGGTACCGCCAAGCGC-3′, and F187T_reverse: 5′-GCGCTTGGCGGTACCGAAGTC-3′.

### 2.2. Western Blotting

The autophosphorylation status of recombinant PKA Cα wild type (wt) and F187V at position T197 and S338 was investigated using Western blot analysis. Purified proteins were denatured in SDS sample buffer and loaded onto SDS polyacrylamide gels. The transfer on a nitrocellulose membrane was performed utilizing a semi-dry transfer system. For visualization, we used the polyclonal rabbit IgG antibodies Phospho-PKA alpha/beta α-pT197 (44-988A; Cell Signaling Technology, Danvers, MA, USA) and Phospho-PKA beta α-pS338 (44-992G; Invitrogen, Thermo Fisher Scientific, Waltham, MA, USA). As a control, the PKA C subunits were detected using an α-PKA-Cα: scFv-Fc-Fusion (YumAb, human Fc region) protein (YumAb GmbH, Braunschweig, Germany). Secondary antibodies used were polyclonal α-rabbit IgG horseradish peroxidase antibodies (Amersham Bioscience, Little Chalfont, UK) and polyclonal α-human IgG horseradish peroxidase antibodies from goat (Sigma-Aldrich, St. Louis, MO, USA).

### 2.3. Spectrophotometric Kinase Assay

To determine the Michaelis-Menten constant (K_M_) and the turnover number (k_cat_) of purified PKA Cα wt and the DFG+1 mutants for the peptide substrate Kemptide, a coupled spectrophotometric assay was used [38]. As we were interested in the substrate specificity of the kinase, we tested two different peptide substrates: S-Kemptide (LRRA**S**LG) as a serine substrate and T-Kemptide (LRRA**T**LG) as a threonine substrate (GeneCust, Boynes, France). 50 nM PKA Cα wt were used when measured with T-Kemptide and 20 nM wt, F187I, or F187T when measured with S-Kemptide. In all other assays, the final kinase concentration was 10 nM of the respective kinase. All kinases were measured with a minimum of three independent replicates. The calculated turnover was plotted against the kinase concentration and analyzed with GraphPad Prism 8.0 (GraphPad Software, San Diego, CA, USA).

### 2.4. Phosphospecific Antibody-Based Kinase Assay

In vitro kinase assays were performed in 200 μL reactions containing 20 mM MOPS, pH 7.0, 150 mM NaCl, 0.1 mM ATP or 0.2 mM AMP-PNP (adenylyl-imidodiphosphate), 1 mM MgCl_2_, and 1.5 μM substrate protein (GST-PKS or GST-PKT). The reaction was started by adding the kinase to a final concentration of 0.25–1.5 μM. The reaction was stopped after 5 min by adding 2× SDS sample buffer. The samples were loaded onto SDS polyacrylamide gels and transferred to a membrane for Western blot analysis using either a phospho-PKA substrate antibody (α-RRXS*/T*; 100G7E, monoclonal rabbit IgG, Cell Signaling Technology, Danvers, MA, USA) or a polyclonal α-GST antibody (3998.1; Carl Roth, Karlsruhe, Germany). For visualization, an IRDye 800CW donkey α-rabbit IgG secondary antibody (LI-COR, Lincoln, NE, USA) or a polyclonal α-rabbit IgG horseradish peroxidase (Amersham Bioscience, Little Chalfont, UK) antibody were used.

### 2.5. Radioactive Kinase Assay

A radioisotopic kinase assay was performed as previously described following in principle the method by Kish and Kleinsmith [35,39]. Briefly, the reaction mixture of 300 µl contained 30 µM GST-PKS or GST-PKT, and approximately 550 fmoles [γ-^32^P]-ATP (stock solution 110 TBq/mmol, HARTMANN ANALYTIC GmbH, Braunschweig, Germany) in 20 mM MOPS, pH 7.0, 150 mM NaCl, 0.1 mM ATP, 1 mM MgCl_2_. The reaction was initiated by adding PKA Cα to a final concentration of 5 nM. The mixture was incubated with shaking at 30 °C and 350 rpm. Samples of 50 µl were taken after 20, 40, 60, and 80 min and mixed with 500 µl ice-cold ATP buffer solution (20 mM MOPS, pH 7.0, 150 mM NaCl, 1 mM ATP). Instantly, proteins were precipitated by adding 550 µl ice-cold 10% trichloroacetic acid (TCA) plus 3% sodium pyrophosphate. The samples were sucked through mixed cellulose ester membrane filters (MF-Millipore Membrane Filter, 0.45 μm, 25 mm diameter) presoaked in 1 mM ATP for 30 min. Each filter was washed twice with 5 mL of ice-cold 5% TCA containing 1.5% sodium pyrophosphate. Radioactivity of each filter was counted in 20 mL of distilled water in a scintillation counter (Hidex 300 SL; Hidex, Turku, Finland), for 300 s or until a maximum of 10,000 counts was achieved) by detection of Cerenkov radiation. For each run, the total amount of radioactivity, and the blank (reaction mixture without kinase) was measured. For evaluation, all values (cpm) were blank value subtracted. Data evaluation was carried out using GraphPad Prism 8.0 (GraphPad Software, San Diego, CA, USA).

### 2.6. Surface Plasmon Resonance (SPR)

SPR-based interaction analyses were performed using a Biacore T200 instrument (Cytiva, Marlborough, MA, USA) as previously described [35]. A polyclonal α-GST antibody (3998.1; Carl Roth, Karlsruhe, Germany) was immobilized to a CM5 sensor chip (S-series; Cytiva, Marlborough, MA, USA) surface by custom amine coupling. All measurements were performed at 25 °C with a flow rate of 30 µl/min using running buffer (20 mM MOPS, pH 7.0, 150 mM NaCl, 0.1 mM EDTA, 0.01% P20 surfactant) supplemented with nucleotides and metal ions as indicated. In each measurement cycle, about 50 RU of GST fusion protein (GST-PKS, GST-PKT, or GST-PKI) was captured, before PKA Cα was injected for 150 s or 300 s (association). For the analysis of the product complex, GST fusion proteins prephosphorylated with PKA Cα (GST-pPKS or GST-pPKT) were captured [36]. The dissociation phase was induced by switching to running buffer for 150 s or 300 s. SPR signals from a blank flow cell with only α-GST antibody immobilized as well as blank runs with buffer injections were subtracted (double referencing). The sensor chip surface was regenerated by injection of 10 mM glycine, pH 1.9 until the baseline level was reached. Binding kinetics were analyzed with Biacore T200 Evaluation Software 3.0 and BIAevaluation 4.1.1 (Cytiva, Marlborough, MA, USA) applying a 1:1 Langmuir binding model, global fit, or steady-state analysis.

On-chip phosphorylation was performed as previously described [36].

### 2.7. Docking Simulations

Structural models of PKA Cα wt and F187V in complex with AMP-PNP, Mg^2+^, and either SP20 (serine-containing substrate peptide corresponding to the amino acid residues 5–24 of human PKIα isoform with the two mutations N20A and A21S; TTYADFIASGRTGRRA**S**IHD [40]) or TP20 (SP20 with S21T mutation; TTYADFIASGRTGRRA**T**IHD), were generated using the crystal structure of the murine myristoylated PKA Cα, in complex with SP20 and AMP-PNP (PDB code: 4DG0) [25]. The respective amino acids were exchanged in YASARA (V.18.12.27) [41] and the side chain conformations were optimized using the SCWALL approach [42] employing the SCWRL3 algorithm [43] before surface interactions and solvation energies were optimized using the YASARA2 force field [42]. The structures were subsequently disassembled into SP20 or TP20 and either PKA Cα wt or PKA Cα F187V. Energy minimization was performed with the (em_run) macro of YASARA [41], employing the Amber14 forcefield [44] and the TIP3P water model [45]. Docking of SP20/TP20 to PKA Cα wt/F187V was performed with the dock_runlocal macro [41,46] which employs a YASARA-specific version [41] of AutoDock VINA [47]. In this process, a simulation cell with a side length of x ≈ 44.185 Å, y ≈ 38.491 Å, and z ≈ 38.882 Å was placed around the SP20/TP20 and the binding site of PKA Cα wt/F187V. To further optimize the conformation of all elements within the simulation cell, the NOVA force field [46] was utilized by the dock_runlocal setup. The YASARA AutoSMILES algorithm was used for simulations with a derivative of MOPAC [48] and the COSMO solvation model [49] before calculating and adding AM1BCCs [50] and applying the GAFF [51] to generate all values unknown to the NOVA force field [41,46,52]. Values for divalent metal ions were taken from [53]. After the docking was performed, each generated model was again submitted to the em_run macro [41].

## 3. Results

### 3.1. The DFG+1 Residue Determines the Serine Specificity of PKA

Early peptide studies based on radioactive kinase assays have already demonstrated that PKA prefers serine over threonine substrates [20]. Using a spectrophotometric kinase assay, we recapitulated that PKA Cα phosphorylates the peptide substrate S-Kemptide with lower K_M_ and higher k_cat_ compared to T-Kemptide (Table 1, Figure S1) [20,29]. This leads to a more than 100-fold higher catalytic efficiency (k_cat_/K_M_) for the serine substrate peptide. To test the role of the DFG+1 phenylalanine (F187) for Ser/Thr specificity, we mutated F187 to valine, isoleucine, and threonine, since β-branched amino acids at this position were postulated to mediate threonine specificity [28]. Indeed, changing the DFG+1 position resulted in higher catalytic efficiency for T-Kemptide compared to S-Kemptide (Table 1). This is partly because the k_cat_ values for the threonine substrate were at least 5-fold increased and exceeded the k_cat_ of PKA Cα wt for S-Kemptide. Interestingly, the mutation F187V also increased the k_cat_ for phosphorylation of S-Kemptide compared to the wild type. Additionally, mutating the DFG+1 phenylalanine residue reduced the K_M_ for T-Kemptide about 30-fold (Table 1). While K_M_ values for S-Kemptide were increased 4-fold for both PKA Cα F187V and F187I, the K_M_ for PKA Cα F187T remained unaffected.

### 3.2. PKA Cα Phosphorylates PKT Faster than PKS

To study the substrate specificity of the PKA Cα subunit on protein substrates, we used PKI-derived serine (PKI A21S, PKS) and threonine (PKI A21T, PKT) substrates. Previously, we have shown that PKS is a high-affinity substrate of PKA [36].

**Figure 2:**
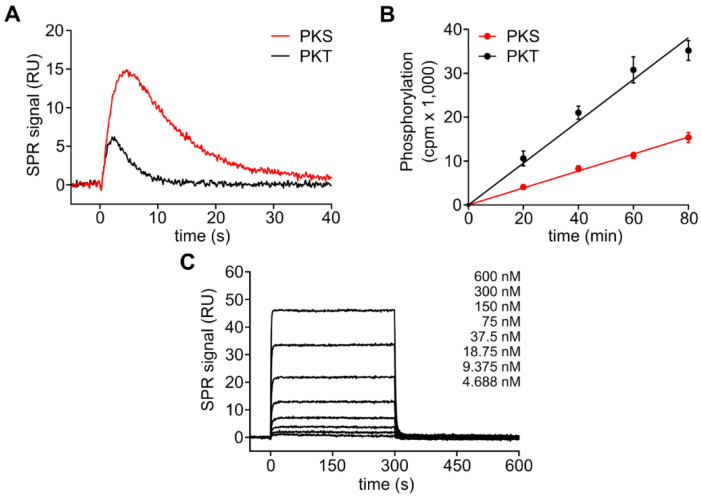
Phosphorylation of the threonine substrate PKT. (**A**) On-chip phosphorylation of GST-PKS (red) and GST-PKT (black) with 100 nM PKA Cα wt (Figure S2). On-chip phosphorylation of GST-PKT is faster than phosphorylation of GST-PKS. Equal amounts of GST-PKS and GST-PKT were immobilized to two parallel flow cells to analyze on-chip phosphorylation by PKA Cα simultaneously. (**B**) Radioactive kinase assay. Substrate phosphorylation with radioactively labeled γ-^32^P-phosphate was measured over time. Data were plotted as means of two independent protein preparations that were each measured in duplicate with standard deviation (SD). Phosphorylation of GST-PKT (black) was twice as fast as phosphorylation of GST-PKS (red). (**C**) SPR analysis of the product complex with immobilized GST-pPKT in the presence of 0.2 mM ADP and 1 mM MgCl_2_. See Table 2 for kinetic data.

First, we tested if PKA Cα was able to phosphorylate PKT in vitro. Using a phospho-PKA substrate antibody (α-RRXS*/T*), we found that GST-PKT (PKT) was strongly phosphorylated by PKA Cα at T21 in the presence of MnCl_2_ (Figure S2). We next employed an SPR-based on-chip phosphorylation assay [36]. In this setup, a substrate (GST-PKS or -PKT) is captured on a sensor chip and by injecting the PKA C subunit, complex formation, as well as dissociation, are monitored in real-time. The resulting SPR curve reflects an overlay of several events happening during the time course of the measurement. Fast substrate-enzyme complex formation together with fast dissociation of the product is signified by a sharp peak, while peak broadening indicates a slower dissociation of the product. While this approach cannot be used to determine reaction velocities (since [E] >> [S]), on-chip phosphorylation signals can give a hint on the kinetics of the phosphorylation reaction. Interestingly, in the SPR on-chip phosphorylation assay, PKT gave a sharp signal peak which was narrower compared to the peak for PKS indicating faster phosphorylation of the threonine substrate (Figure 2A, Figure S3). The faster turnover of PKT compared to PKS was confirmed in a kinase assay employing γ-^32^P-ATP (Figure 2B). Thus, PKT is—to the best of our knowledge—the first known PKA substrate where the exchange of the phosphoryl acceptor from serine (PKS) to threonine increases the speed of phosphorylation.

As product release is often the rate-limiting step in kinase catalyzed reactions, we next tested if the faster turnover of PKT was due to a faster dissociation of the product complex (PKA Cα:Mg_2_:ADP:pPKT). We, therefore, compared the interaction of the C subunit with phosphorylated GST-PKT (pPKT) in the presence of ADP and Mg^2+^ with our previously published data for pPKS [36]. The dissociation of the C subunit was at least 6-fold faster for pPKT than for pPKS, which resulted in a 9-fold higher K_D_ for the phosphorylated threonine product (Figure 2C, Table 2). This indicates that indeed product release is the rate-limiting step and thus faster dissociation of pPKT leads to increased turnover of PKT compared to PKS. Yet, since the phosphorylation of PKT was only twice as fast as for PKS, other steps of the catalytic cycle may be slowed down with a threonine phosphoryl acceptor (Figure 2B).

**Figure 3:**
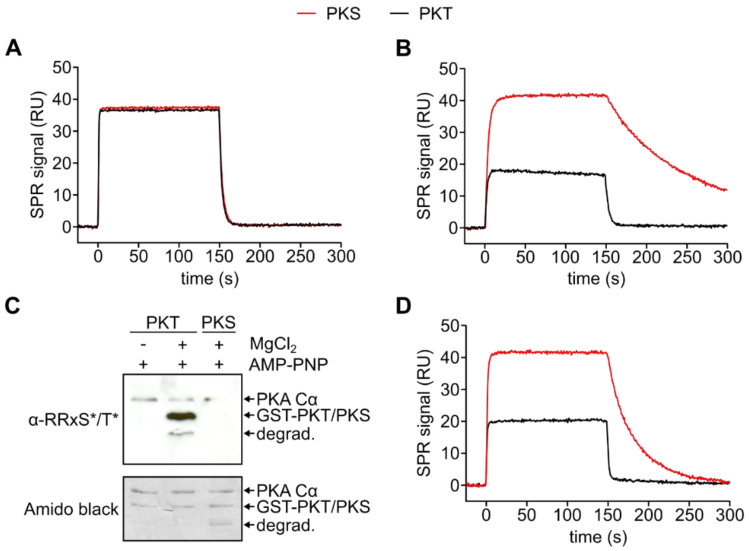
SPR analysis of the formation of the Michaelis complex. (**A**) Comparison of the interaction between PKA Cα (900 nM) and GST-PKS (red) and GST-PKT (black), respectively, in the absence of metal ions and nucleotides (Figure S4). Both binding kinetics are similar. (**B**) Comparison of the interaction of PKA Cα (100 nM) with GST-PKS (red) and GST-PKT (black), respectively, in the presence of 0.2 mM AMP-PNP and 1 mM MgCl_2_ (Figure S5). The Michaelis complex with PKS is stabilized while in the case of PKT, the complex dissociates faster. (**C**) Western blot with a phospho-PKA substrate antibody (α-RRXS*/T*). Phosphorylation of GST-PKT by PKA Cα can be detected after 5 min incubation at 25 °C in the presence of 0.2 mM AMP-PNP and 1 mM MgCl_2_. (**D**) Interaction of 300 nM PKA Cα with GST-PKS (red) and GST-PKT (black) in the presence of 0.2 mM AMP-PCP and 1 mM MnCl_2_ (Figure S6). The complex with PKS can be stabilized with Mn^2+^ ions, while the PKT Michaelis complex dissociates fast.

Again employing SPR, we measured the kinetics of the formation of the enzyme-substrate complex. We could demonstrate that in the absence of metal ions and nucleotides, the binding kinetics of GST-PKS (PKS) and PKT are identical (Figure 3A). To analyze the (pseudo-)Michaelis complex, we used AMP-PNP, in theory, a non-hydrolyzable ATP analog, as a cosubstrate. PKT showed at least 45-fold lower affinity to the C subunit compared to PKS, which was due to a faster dissociation (Figure 3B, Table 3). Interestingly, the SPR signal during the association of PKA Cα to PKT did not reach a binding equilibrium but decreased slightly (Figure 3B). As it was previously shown that AMP-PNP can serve as a phosphoryl donor under specific circumstances, for example within a protein crystal [54], this indicates phosphorylation of PKT. We, therefore, tested if PKA Cα can use AMP-PNP for substrate phosphorylation during the time course of an SPR injection (300 s). We found that PKT, but not PKS, was phosphorylated in the presence of AMP-PNP and MgCl_2_ (Figure 3C). This shows that the Michaelis complex between PKA Cα and PKT is less stable than the complex between PKA Cα and PKS. However, PKT is still a better substrate as indicated by its phosphorylation in the presence of AMP-PNP.

To abolish phosphorylation of PKT during interaction analysis, another non-hydrolyzable ATP analog, AMP-PCP (adenylyl-methylenediphosphonate), was utilized (Figure 3D). As a phosphonate AMP-PCP is not hydrolyzed by PKA Cα (Figure S2). Measurements with AMP-PCP and Mg^2+^ showed a fast dissociation of the substrate which could not be resolved with SPR measurements (Figure S7). In previous studies, we could demonstrate that AMP-PNP and Mn^2+^ displayed a slower substrate dissociation when compared to Mg^2+^ [35]. Based on these results, we used Mn^2+^ here again to stabilize the Michaelis complex (Table 4). Dissociation of the complex with PKT was 14-fold faster compared to PKS leading to a 22-fold lower affinity for the threonine substrate.

### 3.3. Mutation of the DFG+1 Residue Switches the Ser/Thr Specificity of PKA Cα

**Figure 4:**
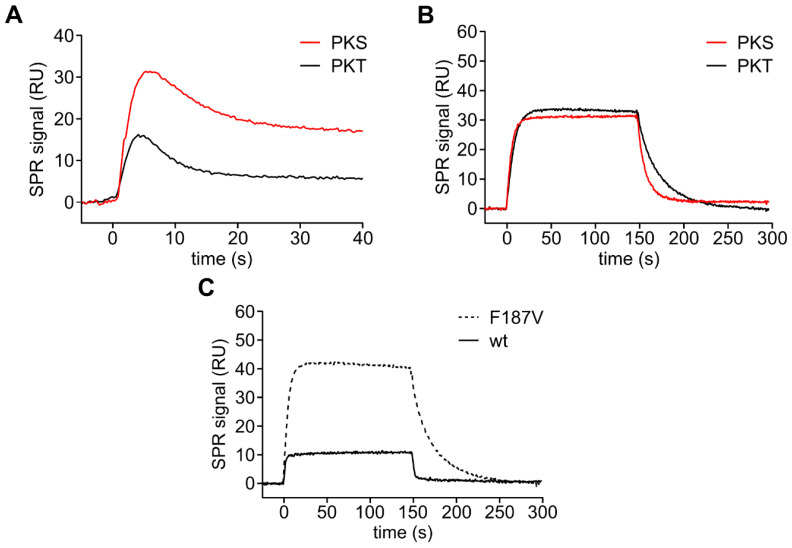
PKA Cα F187V has a higher affinity for the threonine substrate PKT. (**A**) On-chip phosphorylation of GST-PKS (red) and GST-PKT (black) with 100 nM PKA Cα F187V (Figure S9B,C). PKA Cα F187V phosphorylates GST-PKT faster than GST-PKS. (**B**) SPR analyses of the interaction between 50 nM PKA Cα F187V and GST-PKS (red) and GST-PKT (black), respectively, in the presence of 0.2 mM AMP-PCP and 1 mM MnCl_2_ (Figure S9D,E). The mutant shows a slightly slower dissociation for the threonine substrate PKT. (**C**) Comparison of the binding curves of PKA Cα wt (solid line) and F187V (dashed line; 100 nM each) demonstrates that the mutant has a higher affinity for the threonine substrate PKT as the wt. See Table 4 for the determined rate constants and K_D_ values.

As we and others have shown before that mutating the DFG+1 residue increases the turnover of threonine peptide substrates by PKA Cα [28], we asked how the amino acid exchange F187V affects the phosphorylation and the affinity of PKT. Recombinantly expressed PKA Cα F187V could be purified using PKI affinity chromatography although its affinity to the heat-stable protein kinase inhibitor was 10-fold reduced (Figure S8; k_a_ = 3.6 × 10^6^ M^−1^s^−1^; k_d_ = 2.0 × 10^−2^ s^−1^; K_D_ = 5.6 nM) compared to the wild type protein (k_a_ = 1.5 × 10^6^ M^−1^s^−1^; k_d_ = 0.077 × 10^−2^ s^−1^; K_D_ = 0.5 nM) as previously reported [55]. The recombinant protein was an active protein kinase as indicated by autophosphorylation at threonine 197 and serine 338 (Figure S9A). PKA Cα F187V showed faster on-chip phosphorylation of PKT indicated by a narrower association peak compared to PKS (Figure 4A). Again, we analyzed the kinetics of the formation of the Michaelis complex in the presence of AMP-PCP and MnCl_2_ to avoid phosphorylation of the substrates. PKA Cα F187V bound PKT with a more than 2-fold higher affinity compared to PKS due to a slower dissociation (Figure 4B, Table 4). F187V has an almost 10-fold higher affinity for the threonine substrate compared to wt caused by a 10-fold slower dissociation (Figure 4C, Table 4). Thus, the sterically less demanding valine residue slows down the dissociation of the threonine substrate PKT.

Taken together, we demonstrate that the pronounced serine specificity of the PKA catalytic subunit is based on a lower affinity towards threonine substrates. Our comparative analysis of PKA Cα wt and F187V underlines that the DFG+1 motif is a crucial determinant for this specificity.

## 4. Discussion

cAMP-dependent protein kinase is the main receptor of the second messenger cAMP and plays a critical role in various signaling pathways controlling energy metabolism, neurophysiology, and endocrine response. Mutations in PKA isoforms have been considered rare, however, recently several disease phenotypes have been directly attributed to mutations either on the PKA C or R subunits [3,56,57]. As the first protein kinase whose crystal structure was solved [58], the function of PKA has been investigated in detail for decades. It has already been shown that PKA phosphorylates serine peptide substrates more efficiently than threonine substrates [18,20,59] and recently mutational analysis revealed the DFG+1 residue as a major determinant for Ser/Thr specificity [28]. Yet a detailed study uncovering the underlying mechanisms has been lacking so far.

Employing a multi-tiered approach, we investigated individual steps of the catalytic cycle comparing serine and threonine substrates side by side. We used Michaelis–Menten kinetics to analyze the phosphorylation of substrate peptides and subsequently studied the kinetics of the Michaelis complex and the product complex with protein substrates. Based on a well-established peptide substrate, the heptapeptide Kemptide, we could again show that PKA has a strong preference for serine versus threonine substrates. Replacing the critical DFG+1 phenylalanine (F187) with any β-branched amino acid (F187 to I/T/V) switched the Ser/Thr-specificity of PKA confirming the prediction of Chen and coworkers [28].

The protein substrates (PKS and PKT) were investigated by a combined approach employing radioactive kinase assays and SPR binding studies. The binding curves for serine and threonine substrates were similar in the absence of nucleotides and metal ions reflected in fast on- and off-rates. Those data are comparable to data for the PKA Cα:PKI complex without nucleotides and metal ions [55] displaying an affinity in the higher nM range (GST-PKI:PKA Cα: K_D_ = 450 nM [55], GST-PKS:PKA Cα: K_D_ = 124 nM; GST-PKT:PKA Cα: K_D_ = 167 nM; Figure S4). It has been demonstrated that ATP and two divalent metal ions are required to achieve a high-affinity complex between the catalytic subunit and the pseudosubstrate inhibitor PKI [55,60]. In this conformation, the catalytic subunit is locked in the fully closed state. During the catalytic cycle, the kinase needs to toggle between open and closed conformations, which involves, in particular, the glycine-rich loop [36,61]. The role of metals and nucleotides for the closing of the glycine-rich loop has been demonstrated in different crystal structures and is reviewed in [61]. Hence, we speculate that PKA C subunits in the open conformation (i.e., without metal and nucleotide) do not discriminate between serine or threonine phosphoryl acceptors. SPR revealed that in the presence of metal ions and adenine nucleotides, PKA Cα binds threonine substrates (here PKT) with lower affinity compared to serine substrates (PKS). Exchanging the large phenylalanine residue in the DFG+1 position (F187) for a smaller valine residue (PKA Cα F187V) increased the affinity for the threonine substrate by reducing the off-rate, underlining the importance of this position for substrate specificity. Based on K_M_ values and crystal structures of PAK4 wt and PAK4 F461V in complex with PAKtide-S and PAKtide-T it was assumed that threonine and serine peptides bind with similar affinities [28]. Decreased phosphorylation of threonine substrates was attributed to conformational restrains in the catalytic center such as the DFG+1 phenylalanine residue. This was also extrapolated to the catalytic subunit of PKA. While the PAK4 crystal structures depict the importance of the orientation of the phosphoryl acceptor, no nucleotide or metal was visible in most structures with PAKtide, indicating that the kinase was not in the fully closed conformation [28]. At least for PKA, we unambiguously demonstrate that the preference for serine versus threonine substrates is also reflected in their affinity.

**Figure 5:**
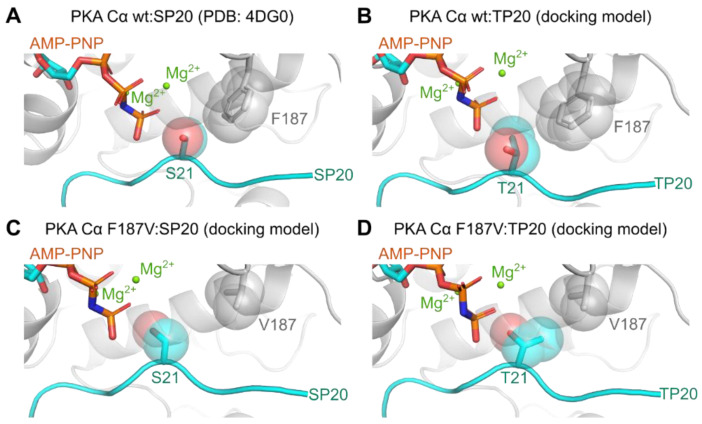
The Ser/Thr specificity of PKA is determined by the DFG+1 motif. (**A**) The binding pocket of PKA Cα in complex with AMP-PNP, Mg^2+^, and SP20 (PDB code: 4DG0) [25]. The DFG+1 phenylalanine (F187) occupies a large space directly adjacent to the P0 site, coordinating the SP20 serine at this position. (**B**–**D**) Model structures derived from docking simulations performed with YASARA (v.18.12.27) [41,52]. (**B**) The hydroxyl group of the threonine at the P0 site (T21 in TP20) points away from the γ-phosphate due to the additional methyl group, which could hinder the phosphoryl transfer. (**C**) In the case of the F187V mutation, the P0 serine can rotate more freely due to the smaller branched valine at the DFG+1 position. (**D**) The valine could allow a favorable orientation of the threonine at the P0 site for phosphoryl transfer. All structure images were generated using the PyMOL Molecular Graphics System (Version 2.2.2; Schrödinger, LLC, New York, NY, USA).

To gain insights into the protein kinase-substrate complex with metal and nucleotide bound to the kinase, we performed docking simulations to generate structural models of the Michaelis complexes of both PKA Cα wt and F187V with PKS and PKT, respectively (Figure 5). As a template, we used the PKA Cα wt:SP20 structure co-crystallized with AMP-PNP and Mg^2+^ (PDB code: 4DG0) [25]. In the crystal structure of PKA Cα wt bound with SP20, F187 seems to limit the available space for the serine side chain, benefiting the orientation towards the AMP-PNP γ-phosphate (Figure 5A). In the case of threonine as phosphoryl acceptor (T21 in TP20), the DFG+1 phenylalanine restricts the rotational freedom of the threonine side chain due to the increased steric demand between the additional methyl group and the benzene ring of F187 (Figure 5B). Exchanging the DFG+1 phenylalanine for a valine residue (PKA Cα F187V) increases the space and thus the freedom of rotation of the phosphoryl acceptor (Figure 5C,D). In the case of the PKA Cα F187V:SP20 complex, the interaction of the S21 side chain with V187 (Figure 5C) is weaker than the interaction with F187 (Figure 5A). This topology could allow the entrance of additional water molecules leading to a less stable Michaelis complex compared to PKA Cα wt [61]. In contrast, in the complex of PKA Cα F187V with TP20, V187 seems to accommodate the P0 threonine and thus restricts free rotation (Figure 5D). In the model for PKA Cα F187V with TP20, the hydroxyl group is perfectly positioned for phosphoryl transfer contrarily to the PKA Cα wt:TP20 complex (Figure 5B,D). As the F187V mutation reduced the affinity for PKS only slightly, the specificity was not switched completely. We, therefore, hypothesize that the DFG+1 position is a rather weak determinant for threonine specificity but strongly promotes serine specificity. Small or β-branched DFG+1 residues facilitate threonine phosphorylation rather than switching the selectivity towards threonine substrates.

One important finding of our study was that PKA Cα phosphorylated PKT faster than PKS, even in the presence of AMP-PNP. While the chemical step of phosphoryl transfer is considered to be fast, the release of the phosphorylated product and ADP-Mg is rate-limiting [32]. Our data clearly show a faster release of pPKT compared to pPKS, which also explains the faster turnover. This demonstrates that threonine substrates are not per se poor substrates of a serine-specific kinase. In line with this, DARPP32 is a good PKA substrate, although DARPP32-derived peptides are not [62]. Like the PKA RI subunits, DARPP32 carries additional arginine residues in positions P-3 and P-4 that may increase substrate affinity. In PKA, the introduction of a threonine residue in the P0 position reduces substrate affinity. In synthetic peptides, the relative importance of single residues is more pronounced compared to protein substrates since fewer contact points are made with the enzyme [63]. Consequently, the exchange of the phosphoryl acceptor from serine to threonine in the heptapeptide Kemptide reduces catalytic efficiency dramatically. In contrast, for the high-affinity (protein) substrate PKS, the exchange of serine to threonine (PKT) increases the turnover by accelerating the product release.

Furthermore, our interaction studies with PKI indicated that the exchange of the DFG+1 phenylalanine to valine drastically reduces the affinity for pseudosubstrates. Remarkably, other kinases like the closely related cGMP-dependent protein kinase, PKG (DFG+1 phenylalanine residue), or protein kinase C (DFG+1 methionine, Figure 1B) also have sterically demanding amino acid residues in the DFG+1 position and thus prefer serine substrates. At this point, we can only hypothesize that this is a general concept for protein kinases regulated by pseudosubstrate inhibition.

### 4.1. Mutations Affecting Specificity in PKA

Mutations in PKA isoforms have been considered rare, however, several disease phenotypes have been directly attributed to mutations either on the PKA C or R subunits [3,56,57]. We have recently shown that mutation of S53 to leucine (S53L) in the glycine-rich loop of the PKA Cβ1 isoform is associated with severe Cushing’s syndrome [57]. Strikingly, PKA Cβ1 S53L has an increased specificity for S-Kemptide compared to T-Kemptide (Figure S10). However, earlier studies have already demonstrated that S53 influences the substrate specificity of PKA [29]. A mutant form of PKA Cα (S53G) exhibits significantly increased turnover of threonine substrates. However, as S53G also increased the turnover of serine substrates, PKA Cα S53G remained serine-specific. Nevertheless, it would be worthwhile testing if the F187V and S53G mutations can have additive effects on the Ser/Thr specificity of PKA. Interestingly, PKG already carries a glycine residue in the position homologous to S53 (G370 in PKG Iα), and a G370S mutation has been recently associated with thoracic aortic aneurysm and dissection (TAAD) [64].

Another mutation leading to Cushing’s syndrome in the Cα subunit of PKA is L205R [56], which disrupts the intramolecular allosteric network [65]. Consequently, PKA C L205R has a decreased catalytic efficiency for Kemptide and phosphorylates non-canonical substrates leading to a dysregulated signaling network in tumor cells [65,66,67].

### 4.2. Mutations Affecting Specificity in Other Kinases

Pathogenic mutations in protein kinases have been associated with a change in Ser/Thr specificity. The exchange between serine and threonine phosphoryl acceptor residues leads to altered cellular signal transduction affecting, for example, mTOR (mammalian/mechanistic target of rapamycin) signaling [68] or glycogen metabolism [69,70].

The leucine-rich repeat kinase 2 (LRRK2), a Ser/Thr kinase, is linked to both familial and sporadic Parkinson’s disease (PD) [71]. In contrast to PKA, LRRK2 prefers threonine over serine substrates, probably due to the β-branched isoleucine at the DFG+1 position (I2020; Figure 1B) [72]. Interestingly, the substitution of this isoleucine to threonine (I2020T) is one of the most common PD-associated mutations in LRRK2 [73]. LRRK2 I2020T phosphorylates serine substrates more efficiently [74]. Since both isoleucine and threonine are β-branched amino acids, it is likely, that the I2020T mutation does not affect Ser/Thr specificity. We revisited this situation in PKA Cα with the mutations F187I and F187T, and, in support of this idea, both mutations resulted in a threonine specificity with similar k_cat_ values for both substrates (Table 1). However, the F187I mutation increased the K_M_ for S-Kemptide 4-fold compared to wt, while the K_M_ remained unaffected by the F187T mutation. The resulting higher catalytic efficiency (k_cat_/K_M_) of F187T could have a strong impact in a cellular context.

Mice heterozygous for the DFG+1 mutation L597V in the proto-oncogene BRAF have reduced body weight and enlarged hearts [75]. In contrast to the well-known mutation V600E, L597V shows only a slightly increased kinase activity depending on the investigated tissue. Mutation of the DFG+1 residue could generally increase the affinity for non-canonical substrates leading to disease conditions like cancer [76].

## 5. Conclusions

To maintain the integrity of intracellular signaling, pathways need to be segregated. In this line, protein kinase networks must be strictly separated based on their substrate specificity. In cellular signaling pathways, protein kinases can not only distinguish between Tyr and Ser/Thr but also between Ser and Thr substrates. This allows for further fine-tuning by phospho-specific binding proteins such as FHA domains, 14-3-3 proteins, and SH2 domains, which specifically bind pThr, pSer, or pTyr, respectively [11,12,13]. Phosphatases have also been shown to have these kinds of preferences [77,78]. The specificity of the cAMP/PKA signaling pathway is ensured on different (regulatory) levels: (1) signaling can be tissue- or cell-specific due to the expression pattern of the involved proteins, (2) scaffold proteins like A-kinase anchoring proteins (AKAPs) contribute to the generation of microdomains and can even sequester substrates to specific intracellular sites, (3) the involvement/crosstalk of different second messengers, like cAMP and cGMP, and (4) the selectivity of the respective effector proteins for either ligand and finally, (5) specificity for downstream effector substrates with the DFG+1 residue as a key determinant for the substrate specificity of Ser/Thr protein kinases.

## Figures and Tables

**Table 1:** The catalytic efficiency of PKA Cα mutants for serine and threonine substrates. k_cat_ and K_M_ values were determined using a spectrophotometric kinase assay (Figure S1). The values are represented as means of at least three independent measurements with standard deviation (SD).

PKA Cα	Substrate	k_cat_ (s^−1^)	K_M_ (µM)	k_cat_/K_M_ (×10^5^ M^−1^s^−1^)
Wt	S-Kemptide	19.8 ± 1.0	19.8 ± 3.9	10.3 ± 1.7
	T-Kemptide	7.5 ± 0.8	861 ± 184	0.09 ± 0.02
F187V	S-Kemptide	35.7 ± 0.7	81.3 ± 27.1	4.8 ± 1.6
	T-Kemptide	39.2 ± 3.7	31.5 ± 16.0	11.3 ± 4.0
F187I	S-Kemptide	25.4 ± 5.8	92.2 ± 3.8	2.8 ± 0.8
	T-Kemptide	43.0 ± 4.7	19.8 ± 3.3	22.1 ± 4.5
F187T	S-Kemptide	15.7 ± 0.3	23.0 ± 3.1	6.9 ± 1.2
	T-Kemptide	37.1 ± 4.5	25.0 ± 3.4	14.9 ± 0.6

**Table 2:** Comparison of the product dissociation of phosphorylated GST-pPKS and GST-pPKT. Kinetic data derived from SPR analyses in the presence of 0.2 mM ADP and 1 mM MgCl_2_.

Phosphorylated Product	k_a_ (M^−1^s^−1^)	k_d_ (s^−1^)	K_D_ (nM)
GST-pPKS ^1^	1.7 × 10^6^	0.065	38
GST-pPKT	1.2 × 10^6^	0.41	342

^1^ Data published in [36].

**Table 3:** Kinetic data for the formation of the Michaelis complex with PKA Cα wt in the presence of 0.2 mM AMP-PNP and 1 mM MgCl_2_. Data derived from SPR analyses (Figure S5) of the interaction between PKA Cα and GST-PKS or GST-PKT, respectively.

Phosphoryl Acceptor	k_a_ (M^−1^s^−1^)	k_d_ (s^−1^)	K_D_ (nM)
GST-PKS	2.7 × 10^6^	0.89 × 10^−2^	3.3
GST-PKT	2.2 × 10^6^	29 × 10^−2^	136

**Table 4:** Kinetic data for the formation of the Michaelis complex with AMP-PCP and MnCl_2_. Data derived from SPR analyses of the interaction between PKA Cα wt or F187V (Figure S9D,E) and GST-PKS or GST-PKT, respectively, in the presence of 0.2 mM AMP-PCP and 1 mM MnCl_2_.

PKA Cα	Phosphoryl Acceptor	k_a_ (M^−1^s^−1^)	k_d_ (s^−1^)	K_D_ (nM)
wt	GST-PKS	2.8 × 10^6^	3.0 × 10**^−^**^2^	11
	GST-PKT	1.7 × 10^6^	41 × 10**^−^**^2^	241
F187V	GST-PKS	1.1 × 10^6^	7.8 × 10**^−^**^2^	71
	GST-PKT	1.7 × 10^6^	4.2 × 10**^−^**^2^	26

## Supplementary Materials

The following are available online at https://www.mdpi.com/2073-4409/9/6/1548/s1, Figure S1: Michaelis-Menten kinetics of PKA Cα constructs, Figure S2: PKA Cα wt phosphorylates GST-PKT efficiently in vitro, Figure S3: On-chip phosphorylation with PKA Cα wt, Figure S4: Binding of PKA Cα to PKI-derived substrates in the absence of metal ions and nucleotides, Figure S5: Formation of the Michaelis complex in the presence of 0.2 mM AMP-PNP and 1 mM MgCl_2_, Figure S6: Formation of the Michaelis complex in the presence of 0.2 mM AMP-PCP and 1 mM MnCl_2_, Figure S7: Steady-state analysis for the substrate binding of PKA Cα wt in the presence of 0.2 mM AMP-PCP and 1 mM MgCl_2_, Figure S8: Interaction of PKA Cα F187V with GST-PKI, Figure S9: Biochemical analyses of PKA Cα F187V, Figure S10: Cushing’s syndrome mutant PKA Cβ1 S53L has increased serine specificity.
