# Supplementary material for: Molecular Basis for Ser/Thr Specificity in PKA Signaling
Source: Cells. 2020 Jun 25;9(6):1548. doi: 10.3390/cells9061548 (PMC7361990; doi:10.3390/cells9061548)
Supplement: Supplementary file 1 [file cells-09-01548-s001.pdf]

cells MDPI

Supplementary Figures

Knape et al. 2020: Molecular basis for Ser/Thr specificity in PKA Signaling

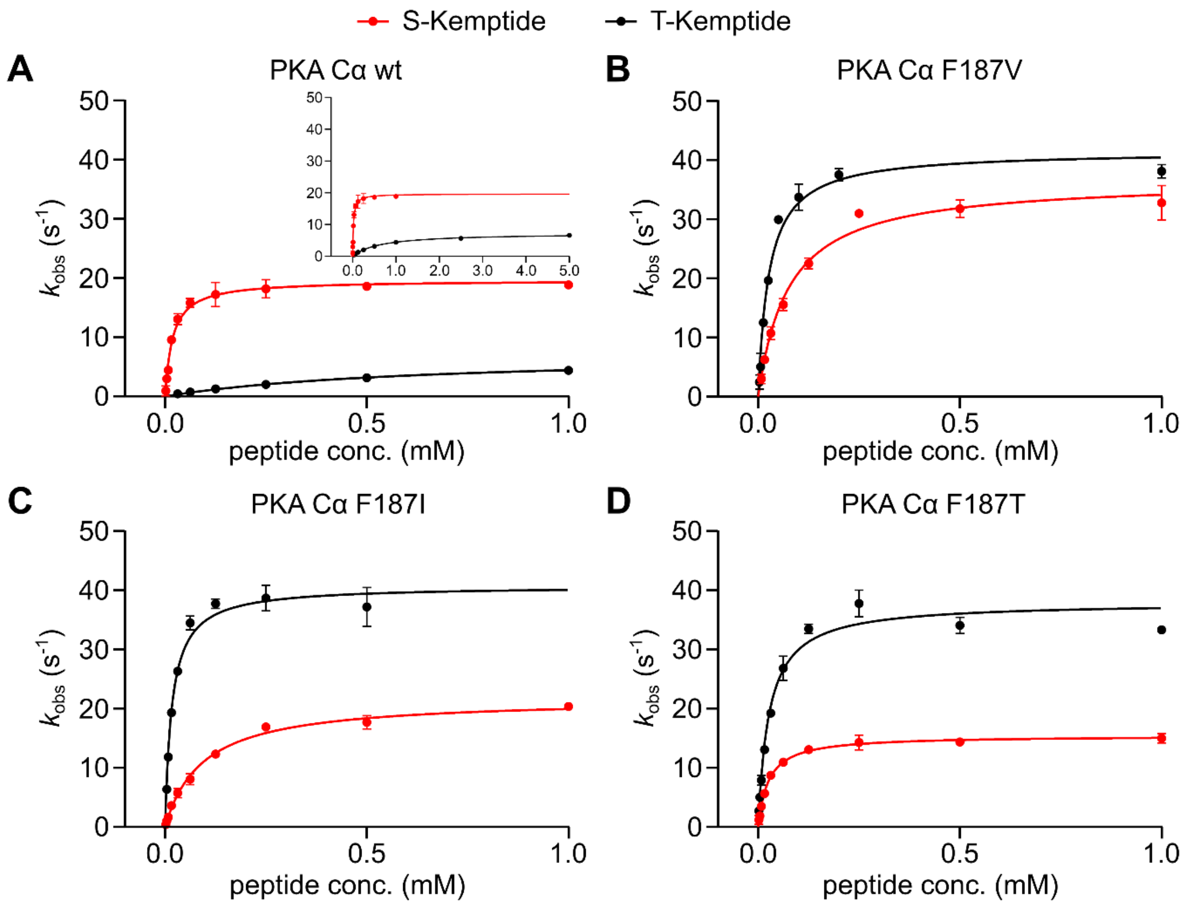

Figure S1. Michaelis-Menten kinetics of PKA C*α* constructs. Phosphorylation of S-Kemptide (red) and T-Kemptide (black) was measured using a spectrophotometric kinase assay. The observed rate constant (k_{obs}) was plotted against the peptide concentration. (A) PKA C*α* wt. (B) PKA C*α* F187V. (C) PKA C*α* F187I. (D) PKA C*α* F187T. The data points are means of two duplicate measurements with error bars indicating the standard deviation (SD). Representative graphs from at least three independent measurements.

cells

MDPI

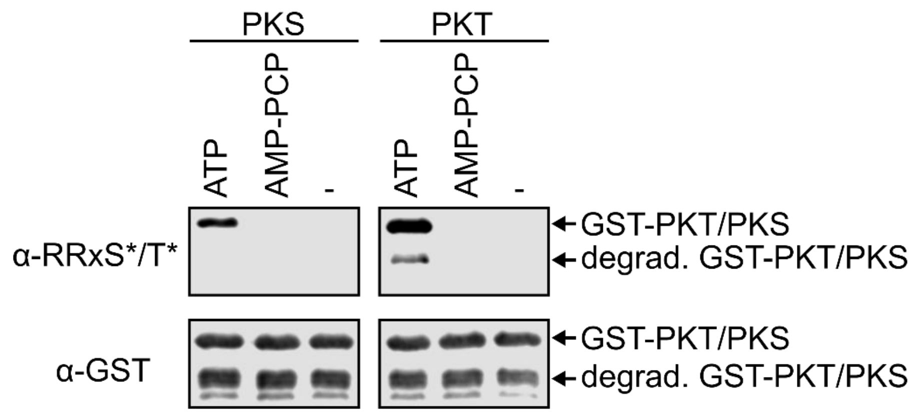

Figure S2. PKA C*α* wt phosphorylates GST-PKT efficiently in vitro. PKA C*α* wt phosphorylates GST-PKS (PKI A21S) as well as GST-PKT (PKI A21T) in the presence of ATP and $MnCl_2$. AMP-PCP and $MnCl_2$ could not be used by PKA C*α* as cosubstrates and showed no phosphorylation of the substrates like in the reactions without any nucleotide. Western blot was performed with a phospho-PKA substrate antibody (*α*-RRXS*/T*) and an *α*-GST antibody as control, respectively.

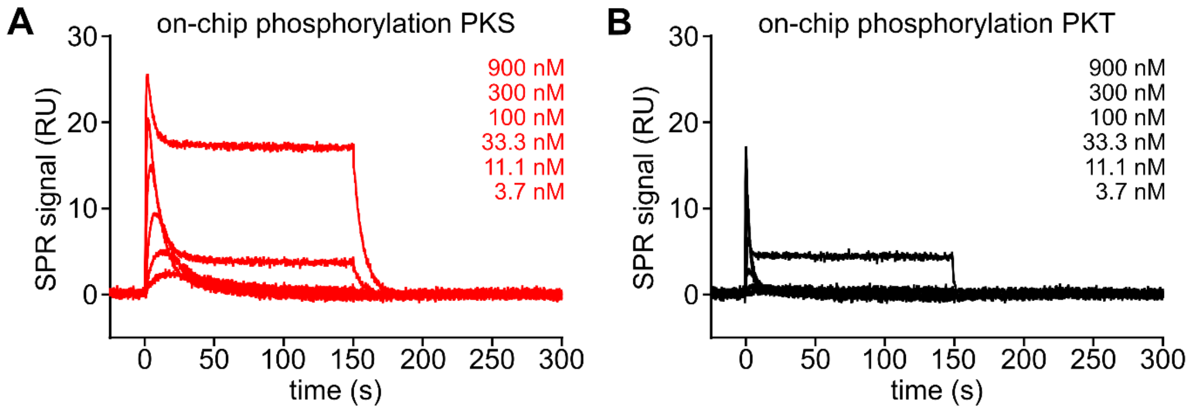

Figure S3. On-chip phosphorylation with PKA C*α* wt. (A) On-chip phosphorylation of GST-PKS. (B) On-chip phosphorylation of GST-PKT.

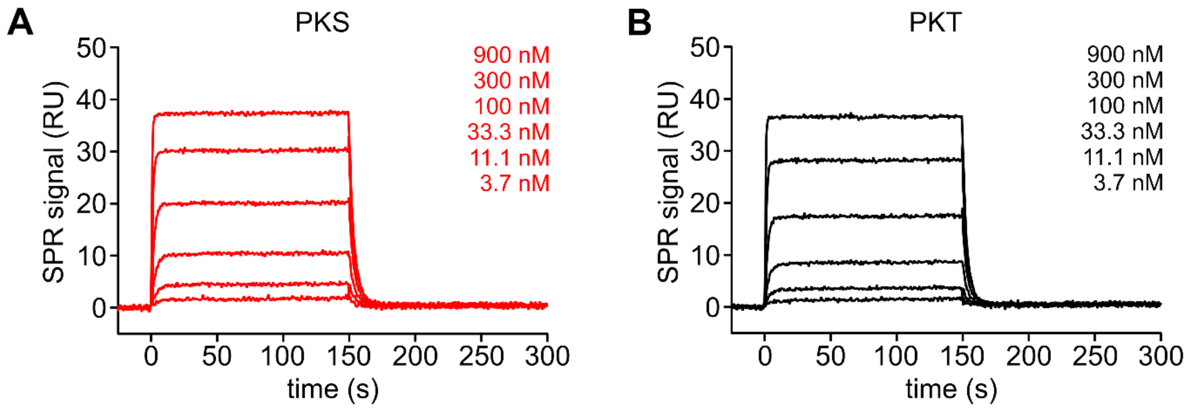

Figure S4. Binding of PKA C*α* to PKI-derived substrates in the absence of metal ions and nucleotides. SPR curves showing the interaction of PKA C*α* wt with (A) GST-PKS and (B) GST-PKT, respectively. The interaction was monitored using a running buffer with EDTA.

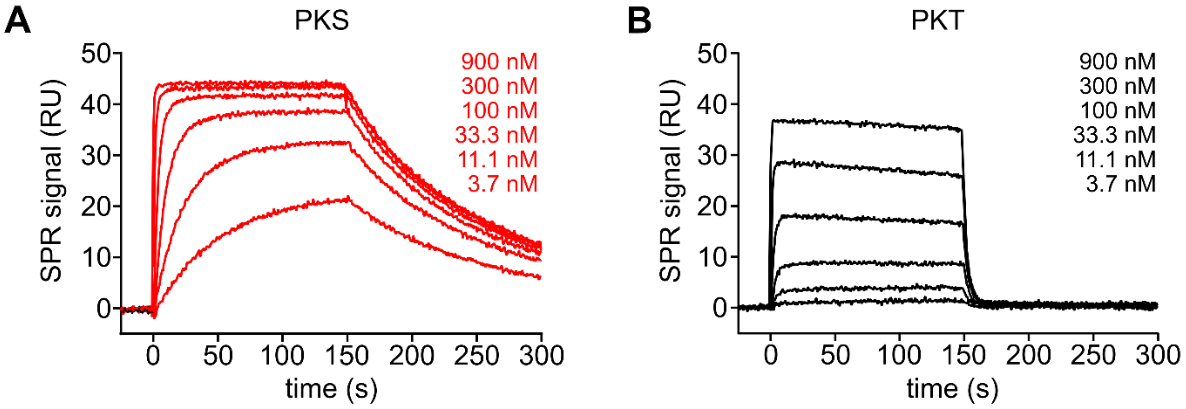

Figure S5. Formation of the Michaelis complex in the presence of 0.2 mM AMP-PNP and 1 mM $MgCl_2$. SPR binding curves showing the interaction of PKA Cα wt with (A) GST-PKS and (B) GST-PKT, respectively.

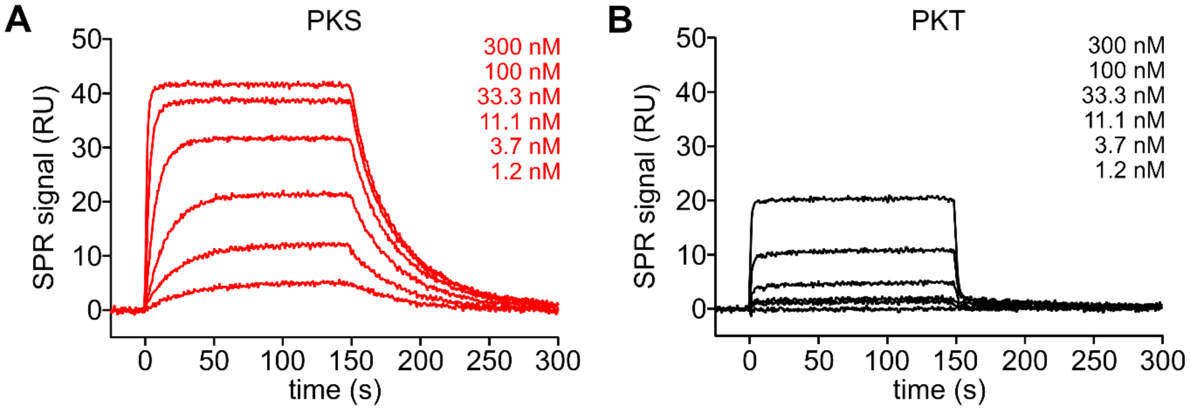

Figure S6. Formation of the Michaelis complex in the presence of 0.2 mM AMP-PCP and 1 mM $MnCl_2$. SPR binding curves showing the interaction of PKA Cα wt with (A) GST-PKS and (B) GST-PKT, respectively.

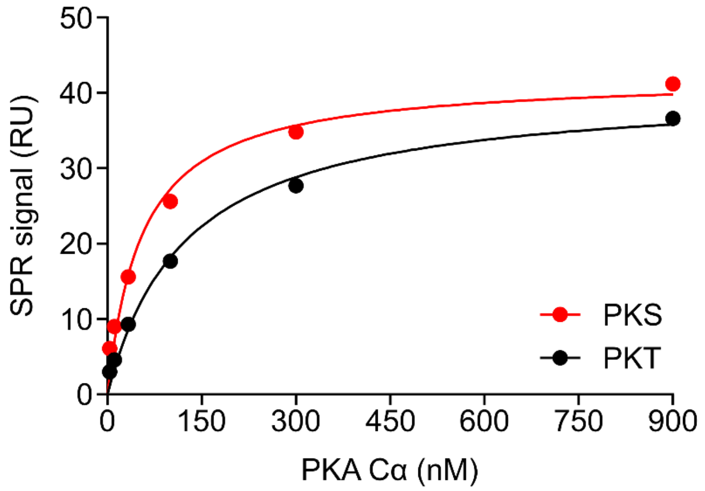

Figure S7. Steady-state analysis for the substrate binding of PKA Cα wt in the presence of 0.2 mM AMP-PCP and 1 mM $MgCl_2$. Equilibrium SPR signals for GST-PKS (red) and GST-PKT (black) were plotted against the respective PKA Cα wt concentration. Determined K_D values for GST-PKS and GST-PKT were 55 nM and 124 nM, respectively.

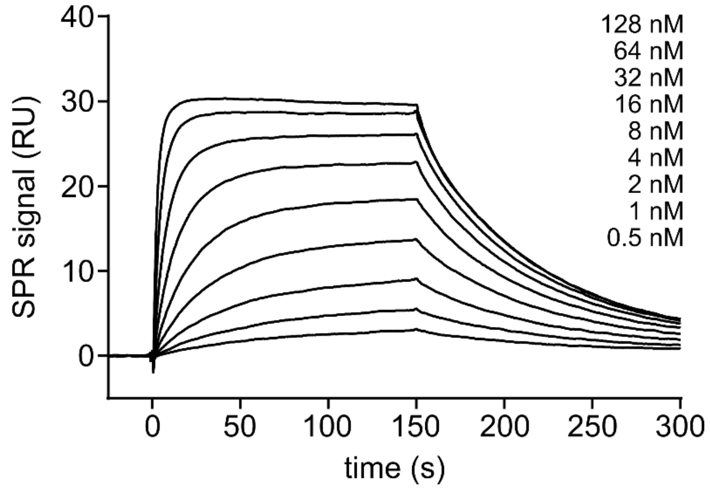

Figure S8. Interaction of PKA Cα F187V with GST-PKI. SPR curves of the interaction analyses in the presence of 1 mM ATP and 10 mM $MgCl_2$.

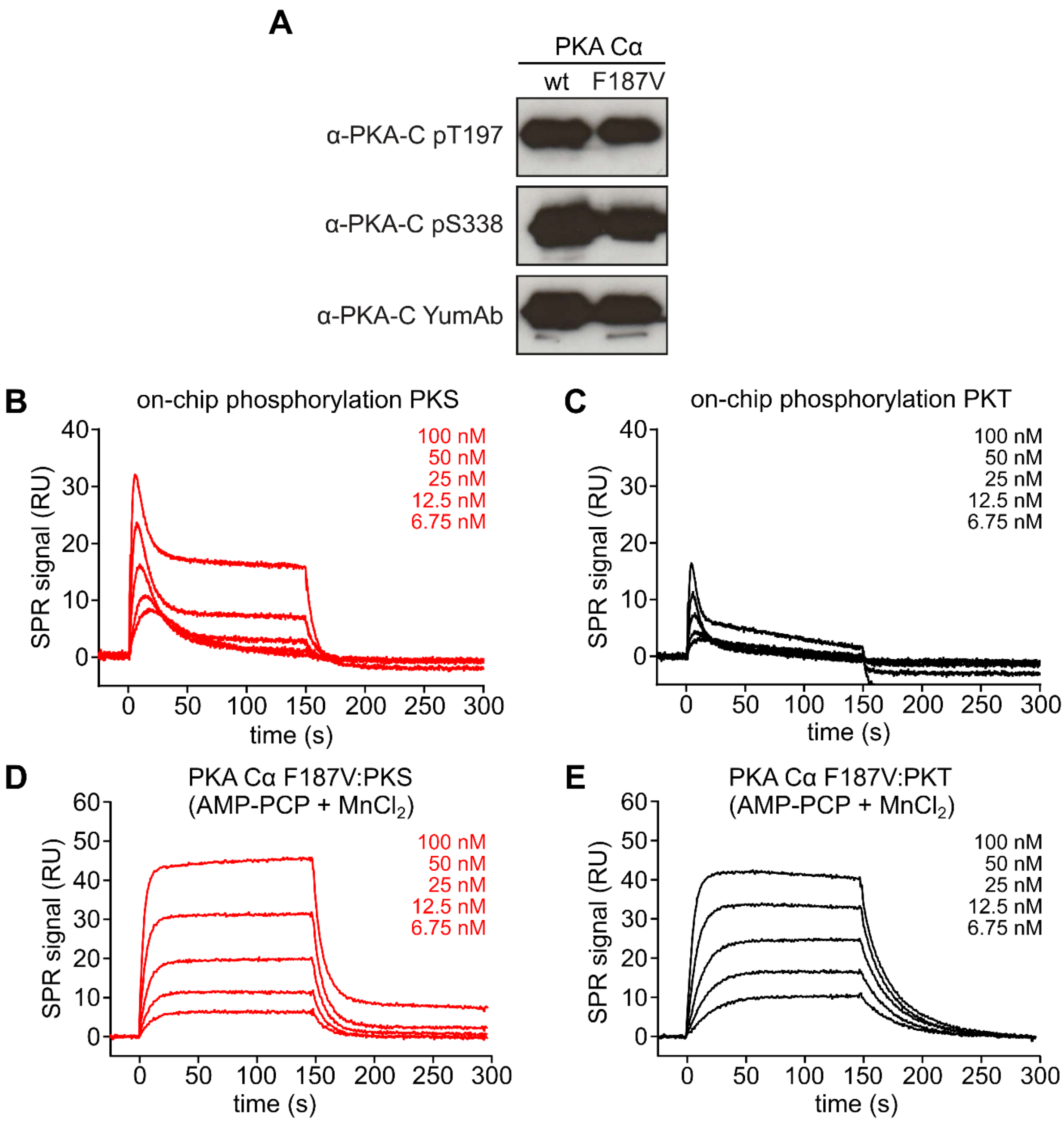

Figure S9. Biochemical analyses of PKA C*α* F187V. (A) Recombinant PKA C*α* F187V is autophosphorylated at T197 and S338 as validated by Western blotting. (B-C) SPR on-chip phosphorylation of GST-PKS (B) and GST-PKT (C) in the presence of ATP and $MgCl_2$. (D-E) SPR binding curves showing the formation of the Michaelis complex between PKA C*α* F187V and PKS (D) or PKT (E), respectively, in the presence of 0.2 mM AMP-PCP and 1 mM $MnCl_2$.

cells MDPI

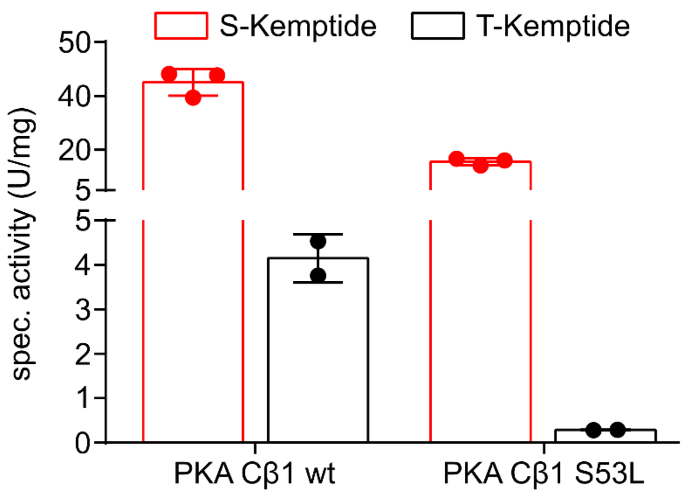

Figure S10. Cushing's syndrome mutant PKA Cβ1 S53L has increased serine specificity. Specific kinase activity of recombinant PKA Cβ1 wt (three independent protein preparations) and S53L mutant (two independent protein preparations) as determined with a spectrophotometric kinase assay using either S-Kemptide (red) or T-Kemptide (black). Bars show the mean values with SD. One unit (U) corresponds to a turnover of 1 µmol substrate per minute.
